# The Role of Social Media in Dental Education: A Qualitative Study

**DOI:** 10.1111/eje.70167

**Published:** 2026-04-17

**Authors:** Amr Elraggal, Stuart Sims, Chris Louca

**Affiliations:** ^1^ University of Portsmouth Portsmouth UK; ^2^ The University of Manchester Manchester UK; ^3^ University of Greenwich London UK

**Keywords:** dental care professional students, dental education, dental students, dental teachers, social media

## Abstract

**Objectives:**

Social media (SM) are now ubiquitous in society. Originally used for socialising between individuals, they are increasingly used for educational purposes. The aim of this study was to explore the role of SM in dental education used by dental students and their teachers in the UK and Egypt.

**Methods:**

Twenty semi‐structured online interviews were conducted with dental students (*n* = 11) and their teachers (*n* = 9) from one UK and two Egyptian universities. The interviews included open‐ended questions about participants' perceptions of using SM in dental education. Data analysis was carried out thematically.

**Results:**

Analysis of the interviews revealed that the participants used SM for communication, collaboration, and to search for information. The dental teachers used SM to promote blended learning and the flipped classroom approach. SM in dental education had many benefits, including ease of use, real‐time communication, and the facilitation of independent, self‐paced student learning. However, some barriers to adopting SM for learning included concerns about the quality of published information, unprofessional behaviour, privacy, and negative effects on learning.

**Conclusion:**

Integrating SM with the traditional classroom approach is potentially useful in promoting an active, collaborative, student‐centred, problem‐based, and blended learning‐oriented environment. However, dental students and their teachers should be trained to use SM appropriately.

## 1 Introduction

Social media (SM) are web‐based platforms that allow users to create personal accounts, add content, and interact with others, share messages, images, audio, or videos by online networking with other users in the same system [1]. SM are dynamic and evolving technology with hundreds of platforms and millions of users. The use of SM is becoming ubiquitous. Initially used as a means of communication and networking between individuals, SM are now increasingly used for educational purposes [2].

SM offer an opportunity for both dental students and their teachers to promote communication and interaction between them in a way that is independent of time or location [3]. This subsequently supports the learning process, allowing it to be collaborative, social and student‐centred [4, 5]. Islam et al. [6] argued that student‐centred learning environments are more effective than teacher‐centred learning environments as they make the classroom more interactive. A teacher‐centred learning environment was believed to be a passive and superficial learning approach [7]. Gow and Kember [8] argued that a student‐centred learning environment is less likely to induce surface learning approaches. Consequently, many researchers report that a transition in curricular and instructional approaches is needed, from teacher‐centred to student‐centred learning environments [9, 10].

SM and associated information and communication technologies help individuals take ownership of their learning [11]. Traditional methods of teaching and learning should be regularly revised and adapted to utilise these technologies within the education system [12].

One challenge of learning through SM is the pressure on students and teachers to keep up with the continually evolving technological changes [13]. In addition, learning through SM is relatively new, and dental schools may not be fully aware of the most effective ways to use these technologies for educational purposes. Moreover, both dental students and their teachers must be familiar with the potential uses and barriers to the use of SM in dental education.

The aim of this study was to investigate:

1. The use of SM in dental education.
2. The benefits of using SM as educational tools.
3. The barriers to using SM in dental education.

## 2 Methodology

Favourable ethical approval was obtained from the University of Portsmouth Science and Health Faculty Ethics Committee (Reference Number: SFEC 2019‐081). For this study, SM were defined as internet‐based platforms that allowed for online informal communication, interaction and the sharing of online materials such as photographs, videos and documents [14].

### 2.1 Study Design

Online semi‐structured interviews were conducted via Zoom at the interviewees' convenience. A semi‐structured interview approach was used, allowing the researcher to use a predetermined list of questions and seek clarification as needed. The study participants included undergraduate dental students and teachers from one (University of Portsmouth Dental Academy) and two Egyptian dental schools (Alexandria University and Pharos University). The participants were invited to take part in this study and provided consent via email.

### 2.2 Semi‐Structured Interview Questions

A semi‐structured interview guide, including open‐ended and prompting questions, was developed and piloted with two dental students and two dental teachers to ensure the questions were comprehensible. The same guide was used, and the same questions were asked of both participating dental students and teachers with minor wording changes. The questions were related to the following main topics in dental education (Table 1):

1. Uses of SM
2. Benefits of using SM
3. Barriers to using SM

**TABLE 1:** Semi‐structured interview questions.

Main themes	Prompting questions
Uses SM for in dental education	Do you use SM for educational purposes? What platforms do you use? How? (if the answer is no, then I will follow up with why instead of how)
	How do you use SM to search for dental information?
	What are the most popular dental topics you look for in SM?
	Do you use SM to communicate with others (teachers, students or other dental professionals) for educational purposes? How?
	Would you prefer communicating with your colleagues/teachers via SM or through more formal methods? Why?
	Do you interact with dental‐related posts published on SM?
Advantages of using SM in dental education	In your opinion, what are the benefits of using SM in dental education?
Barriers of using SM in dental education	What are your main concerns about using SM in learning/teaching?
	In your opinion, what are the limitations of using SM in dental education?

### 2.3 Data Collection

An invitation email was sent to all the dental students and teachers at the participating universities. A convenience sampling technique was adopted in this study [15]. Only individuals willing to participate were included. As a result, 11 interviews were conducted with students and nine with dental teachers. A written informed consent was obtained from each participant before the interviews. All interviews were conducted one‐on‐one and lasted approximately half an hour. The participants were asked open‐ended questions in a neutral tone of voice to minimise researcher influence. Data collection continued until saturation was achieved; that is, until no further insights were elicited from the most recently collected data, and thereafter data collection was halted [16]. No new themes emerged after interview 8 for the students and interview 6 for the teachers. The interviews were conducted in the most convenient language, either Arabic for the Egyptian dental schools or English for the UK dental school, for the participants, as the principal investigator was bilingual and was able to use both languages effectively.

Field notes and digital recordings of all interviews were transcribed verbatim immediately after each interview to enable analysis as data were collected. The interviews conducted in Arabic were translated and transcribed verbatim into English by the same principal investigator. All digital recordings and transcripts were stored on a password‐protected laptop. Participants were not identifiable from either the recordings or the transcriptions of the interviews, and all data were fully anonymised [17].

### 2.4 Data Analysis

Preliminary themes and subthemes were developed and informed by previous studies [5, 18, 19, 20, 21, 22, 23, 24, 25, 26]. Then, the data transcripts and field notes were analysed by the principal investigator using Thematic Framework Analysis [27]. This was conducted in five stages: Familiarisation, Identification of the thematic framework, Coding, Charting and Mapping, and Interpretation. Any additional themes or subthemes not previously identified in the literature that emerged from the data were added to the final framework (Figure 1).

**FIGURE 1:**
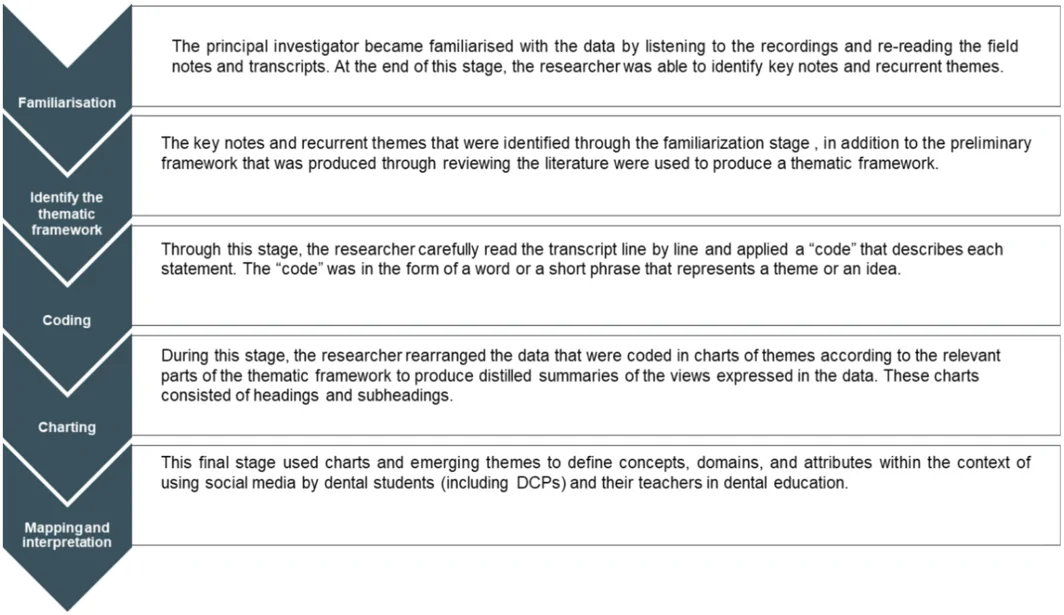
Thematic framework data analysis.

Frequency counts were used to indicate the extent to which each subtheme was represented across participants. After coding the interviews, the number of students and teachers who referred to each theme was recorded. To enable clearer comparison of the relative prominence of themes, these frequencies were converted into a proportional score ranging from 0 to 1, calculated as the number of participants mentioning the theme divided by the total number of participants in that group. This proportional scoring was used descriptively to illustrate the relative weight of themes within the dataset and aligns with recommended approaches for quantifying qualitative data within framework analysis [28].

## 3 Results

A total of 20 semi‐structured online interviews were conducted with dental students (DS) (Egypt X7; UK X4) and their teachers (DT) (Egypt X6; UK X3). Thematic framework analysis revealed three main themes and 15 subthemes. Seven main uses, three main advantages, and 11 main barriers to the use of SM in dental education were identified. Table 2 presents the distribution of themes and subthemes by the number of participants and their frequency counting scores.

**TABLE 2:** Themes and subthemes of data analysis and their frequency counting scores.

Themes	Subthemes	Number of participants			
		DS frequency number (*n* = 11)	DS frequency score (0–1)	DT frequency number (*n* = 9)	DT frequency score (0–1)
Uses of SM in dental education	Students’ collaboration and communication	11	1	7	0.78
	Interaction with other dental professionals	6	0.55	5	0.56
	Using SM for teaching purposes			6	0.67
	Flipped classroom			4	0.44
	Blended learning			6	0.67
	Problem‐based learning			3	0.33
	Information resource	11	1	6	0.67
Advantages of using SM in dental education	Ease of use and accessibility	9	0.82	5	0.56
	Quick way of communication	9	0.82	4	0.44
	Learning at students' own pace	6	0.55		
Barriers of using SM in dental education	Need technical facilities	4	0.36	1	0.11
	The quality of information	9	0.82	7	0.78
	Distracting tools	6	0.55	5	0.56
	Negative effect on learning	5	0.45	3	0.33
	Professionalism	8	0.73	7	0.78
	Posting pictures of patients	8	0.73	7	0.78
	Negative comments	5	0.45	4	0.44
	Unprofessional discussions	4	0.36	3	0.33
	Privacy concerns	8	0.73	6	0.67
	Negative effect on real social life	4	0.36	1	0.11
	The need for motivation	2	0.18	7	0.78

### 3.1 Uses of SM in Dental Education

#### 3.1.1 Students’ Collaboration and Communication

SM platforms, specifically, Facebook, WhatsApp, and Telegram, were used by undergraduate dental students for collaborative purposes, including sharing educational materials like PowerPoint presentations, lecture summaries, handouts, or shared announcements, university updates or information;

we, as year 4, have created a WhatsApp group that is used to share recommendations, announcements, instructions and advice of the tutors. (DS)

Undergraduate dental students in Egypt created Facebook groups to discuss their clinical cases and seek help, without identifying their patients. Many dental teachers were also engaged with students within these groups to deliver advice, help or suggest recommendations when needed;

there is a Facebook group called sharing patients. If someone is looking for a specific clinical case, he will find it through that group. If anyone needs any clinical assistance, they can seek the assistance of that group as well. (DS)

Although universities have created official avenues for online communication between dental teachers and their students, including Email, virtual learning environments (e.g., Blackboard, Moodle), participating dental teachers noticed that students were still looking for unofficial ways to communicate with their teachers;

students usually communicate with me, unofficially, through WhatsApp or Facebook to get (an) assistance or consultation for emergency cases and how to deal with that. (DT)

#### 3.1.2 Interaction With Other Dental Professionals

SM provided an opportunity for both dental students and their teachers to communicate with dental professionals from other regions via SM pages and groups. The participating students and teachers highlighted the value of learning from the posts and discussions conducted on Facebook pages and groups. The participating students also highlighted the value of SM as a forum that connected them with different dental professionals from elsewhere;

for example, with the hygiene and therapy network, …it is like a forum discussion which is educationally helpful, especially to know what other people are think about. (DS)

I follow a couple of dental related Facebook and Instagram pages where I can interact with dental related posts about clinical cases or some new updates in dentistry. (DT)

#### 3.1.3 Using SM for Teaching Purposes

Some participating dental teachers (*n* = 6) shared some educational YouTube videos with their students either before, during or after the classroom lecture to help them understand a technique or a certain topic in a simple way;

absolutely, I use a lot of YouTube clips to demonstrate, especially with things like radiography, physics elements and things like that. (DT)

Some dental teachers mentioned using SM to promote problem‐based learning by posting a case study and asking the students to comment with differential diagnoses, solutions, or treatment plans;

there are some Facebook groups related to the students. Sometimes, we use these groups to post a certain clinical scenario and ask the students to comment with differential diagnosis, or treatment plans. (DT)

#### 3.1.4 Information Resource

Several SM platforms were used as sources of dental information for undergraduate dental students, including YouTube, Facebook, and Instagram;

Instead of looking through large textbooks, we are able to find any piece of information through various SM platforms (DS)

YouTube, for example, was used as the main source of dental information to illustrate practical and clinical dental work. Due to the large number of students in Egyptian dental schools (up to 300 per year), students reported being unable to attend all live demonstrations. YouTube videos were found by dental students to be an effective alternative to missed on‐campus demonstrations and to be useful after graduation for keeping up to date with new technologies in dentistry. Since YouTube is connected to other SM platforms such as Facebook, WhatsApp and Instagram, students were able to share educational material through these groups.

Because of the high number of students watching practical demonstrations at the college, I depend on YouTube videos to watch demonstrations that I missed at the university. (DS)

I do look at videos on YouTube for examples on dental implants to refresh my knowledge. (DT)

### 3.2 Advantages of Using SM in Dental Education

#### 3.2.1 Ease of Use and Accessibility

Study participants highlighted ease of use and accessibility as key advantages of SM. A range of devices, such as personal computers, laptops, and tablets, was available to enable individuals to access their SM accounts seamlessly. In particular, universal access to smartphones among dental students aided them in using SM anywhere and at any time:

SM are easy to use and accessible through many devices like laptops, tablets and smart phones. (DS)

#### 3.2.2 Quick Way of Communication

Most of the participating students and teachers reported that SM, such as Facebook, WhatsApp, and Telegram, enabled rapid communication among dental students and between them and their teachers, bypassing the slow arrangement of in‐person communication. Additionally, SM facilitated group discussions, collaboration, and the sharing of educational materials such as PDF documents, PowerPoint presentations, educational videos, photos, and X‐rays in an easy and rapid way;

………they ease communication and interaction. In addition, they offer a fast way to deliver educational materials like PowerPoint presentations, updates, information, questions and answers. (DS)

it is much easier to pick up your phone and send a WhatsApp message instead of getting a laptop and send structured emails. (DT)

#### 3.2.3 Learning at Students' Own Pace

Six students highlighted the privilege of ‘learning at students’ own pace’ provided by SM. Many dental students' working days are often busy and exhausting. They spend all day moving between classrooms, clinics and laboratories. SM offered them an opportunity to learn at their own pace outside the campus:

…learning through SM gave me the chance to learn in my own space. I definitely would say it is more comfortable. (DS)

### 3.3 Barriers to Using SM in Dental Education

#### 3.3.1 Need Technical Facilities

Some technical issues, such as the quality of the internet connection, cuts in electricity service and problems with equipment, including personal computers, laptops and smartphones, were reported as potential barriers, resulting in some students, particularly at the Egyptian universities, not utilising SM in dental education. This concern was not mentioned by students from the UK.

if we improve some technical issues like the internet connections and electricity interruptions, I will be happy with online learning and will consider learning through SM the same as classroom learning. (DS)

#### 3.3.2 The Quality of Information

Most of the participating students (*n* = 9) and teachers (*n* = 7) were concerned about the accuracy and evidence base of the information published on SM. Moreover, some of the posts found on SM were based on personal opinions that could be biased or incorrect. However, this did not deter dental students from using SM to search for information; they instead turned to their teachers or more evidence‐based resources to assess their accuracy

sometimes you can find incorrect information with no evidence base, wrong or biased opinions (DS)

I have some concerns about YouTube videos, because sometimes they mislead the students by giving them faulty information or techniques. (DT)

#### 3.3.3 Distracting Tools

SM were described by participating students (*n* = 6) and teachers (*n* = 5) as distracting tools, employed through a variety of approaches, such as entertainment videos, games, or online shopping. Also, when using SM such as Facebook or YouTube for educational purposes, it is easy to scroll down and look at some irrelevant videos or posts;

Sometimes, when I go to search for a certain topic, I find myself scrolling and moving from a video to another one and go out of the topic. (DS)

#### 3.3.4 Negative Effect on Learning

One potential negative impact of SM on learning is the risk that they will discourage students from looking for information from traditional resources such as textbooks or reputable journals;

it reduces students’ willing to look for dental information. If someone has a question, he will ask his/her peers instead of looking at the textbooks, scientific papers or the literature themselves. (DS)

The detrimental impact on learning of the sheer volume of information found on SM was also concerning, resulting in information overload and the inability to determine what is correct;

sometimes I find different clinical techniques and I become confused which one is the right, or which techniques should I adopt. (DS)

Two participating teachers reported negative effects of engaging dental students with SM. They argued that some students could be adversely influenced by bad or negative impressions or thoughts stated by others;

when I find someone saying (I hate this subject or dislike that module or topic) they would ask why they should study that. (DT)

#### 3.3.5 Professionalism

Participants referred to some unprofessional uses of SM by dental students, for example, posting clinical photos of their patients;

some students post clinical photos for their patients. I have some doubts if this is ethically accepted or not (DS)

In addition, negative comments and unprofessional discussions have arisen, and this discouraged some students from participating in these discussions;

there was a WhatsApp group for all the level 5 dental therapy, but I, very quickly, came off that. If you disagree with some opinions, or trying to give different perspectives, some of the reactions are really uncalled for, so, I took myself out of that. (DS)

#### 3.3.6 Privacy Concerns

Most of the participating students (*n* = 8) and teachers (*n* = 6) preferred to keep their SM accounts private and separate from the educational process. They were concerned about their privacy and their right to post whatever they wanted on their personal SM accounts. Many students still prefer to communicate with their colleagues or their dental teachers professionally through email and keep their personal SM accounts private;

you have got to be careful what you say on SM, as anything can be misconstrued, and my future career could be in jeopardy if it is taken the wrong way by somebody. (DS)

privacy concerns. I don't want to feel that I am exposed to the rest of the world and everyone can see what I am doing. (DT)

#### 3.3.7 Negative Effect on Real Social Life

Although the main aim of SM is to facilitate communication and socialisation between individuals, it also appears to have had a negative effect on the real social lives of some individuals. Some users have become addicted to the different SM platforms, spending a lot of time in front of their screens talking to others;

they made people sticking to their phones and devices and spending most of their time looking at screens and communication with others through those platforms instead of spending real time in person with friends or families. (DS)

#### 3.3.8 The Need for Motivation

Many participating dental teachers argued that the success of utilising SM in dental education depends mainly on their motivation and willingness to use these platforms. Some dental teachers were motivated more than others;

if teachers were not motivated to be engaged with SM, they wouldn't. (DT)

## 4 Discussion

This study aimed to explore the role of SM in dental education, as used by undergraduate dental students and their teachers. To the best of our knowledge, this is the first qualitative study to investigate both dental students' and teachers' perceptions of the uses, benefits and barriers of SM in dental education. One qualitative study conducted by Sharka et al. [29] focused on the risk factors associated with using SM by dental students and teachers in dental education. However, it didn't explore their educational benefits or their broader applications.

### 4.1 Student‐Centred Learning

The qualitative analysis of the results of this study showed that using SM in dental education enabled student‐directed learning activities, which facilitated student‐centred learning. The resulting positive student experiences confirmed the inclusion of SM to facilitate effective teaching and learning through various interactions [30]. Many studies [23, 30, 31, 32] have highlighted the value of learning through SM in promoting a student‐centred education approach. In a study conducted by Kazi, Saxena and VineetVinay [31], all the participating second‐year dental students reported using Facebook to support independent learning.

### 4.2 Learning Activities

Utilising SM in dental education enhanced learning by facilitating student collaboration, communication, and interaction with other dental professionals, as well as the accessing of information resources.

#### 4.2.1 Student Collaboration

In this study, SM were reported by dental students to be effective tools for collaboration and communication with both their peers and their teachers. This study revealed that dental students used platforms such as WhatsApp, Facebook, and Telegram for academic communication and collaboration. Guraya [33] reported the same observation, stating that SM platforms could facilitate real‐time communication among students outside the classroom, thereby fostering collaborative learning. In addition, SM provided a forum to connect students with their teachers, offering the flexibility of extended learning hours and open and real‐time communication [34].

#### 4.2.2 Information Resource

All the participating dental students used SM, such as YouTube, to search for information. Pre‐recorded YouTube videos were used to supplement practical work in the classroom. These findings were supported by Barry et al. [35] who reported that 78% of medical students used YouTube as their primary source of anatomy‐related video clips, and the same percentage found them either useful or extremely useful for anatomy learning.

This study also demonstrated the popularity of Instagram among participating dental students for short educational videos and photographs that provide brief information on recent techniques and dental innovations [36]. Saadeh et al. [37] argued that the ease of use and ready accessibility of brief information were possible explanations for the preference of undergraduate medical students for SM compared to traditional sources of information, such as textbooks and papers.

#### 4.2.3 Interaction With Dental‐Related SM Posts

This study demonstrated that SM provided dental student participants with the opportunity to interact with posts, thereby facilitating discussions with other followers. SM pages and groups allow for scientific conversations and networking in the form of posts and comments or questions and answers to take place between dental students, their tutors and other dental professionals, which subsequently promote an active learning environment [38].

### 4.3 Learning Environment

This study found that integrating SM in dental education encourages a more social, collaborative, problem‐based, and active learning environment. In addition, it promoted blended learning and the flipped classroom approach.

#### 4.3.1 Social Learning

Using SM by the participants for communication and interaction could facilitate the growth and evolution of knowledge, as reported by Schrader [39]. This can be explained by social learning theory, which posits that new knowledge can be acquired through direct experience or by observing others' behaviour in similar situations. This can explain how social interaction of dental students on SM allows the sharing of implicit knowledge and skills [40, 41].

#### 4.3.2 Collaborative Learning

Collaborative learning was defined as ‘any instructional method in which students work together in small groups towards a common goal’ [42]. In this study, despite the widespread use of formal individualised learning approaches at the participating universities, students often created informal study settings to support their learning. This aligns with findings from another study that revealed that SM tools were used to foster collaboration, interaction, and discussion among dental students [43].

#### 4.3.3 Problem‐Based Learning

The findings of this study revealed that some dental teachers used SM to promote problem‐based learning. SM can foster an active learning environment in which students learn by solving problems [44]. These findings align with another study that reported that approximately 90% of second year medical students found Facebook an effective tool for problem‐based learning [45]. The successful integration of SM has, however, been influenced by teachers' knowledge, experience, approaches to teaching and learning, and information technology literacy skills [44].

#### 4.3.4 Active Learning

The interviewed dental students reported creating their own SM groups in which they communicate, collaborate, ask questions, obtain answers, and debate with one another and with their teachers, to provide a more comprehensive active‐learning experience. SM has the potential to support and enhance active teaching and learning in dental education, providing learners with the necessary tools to access a wide variety of freely available educational content, to create their own study handouts, to connect learners with each other and with their teachers, and to support a collaborative learning environment, as reported in a scoping review conducted by Apel et al. [1].

#### 4.3.5 Blended Learning

The results of this study revealed that all interviewed dental students and six of nine dental teachers used YouTube videos to promote a blended learning environment. Blended learning is a pedagogical mode that integrates traditional face‐to‐face classroom settings along with internet learning activities, allowing the students to create their own learning experience at their own pace and access educational resources from anywhere and at any time [46, 47]. These findings are supported by Aldallal et al. [23] who found that 90% of undergraduate dental students used YouTube videos to learn practical oral surgery procedures independently.

#### 4.3.6 Flipped Classroom

The findings of this study revealed that some dental teachers used SM to promote a flipped classroom teaching approach. The flipped classroom is a form of teaching and learning in which students receive educational materials in advance of and in preparation for the main teaching episode, thereby allowing the teacher to concentrate on in‐depth learning activities in class through actual problem‐solving and clinical exposure [48]. This approach has the advantage of allowing the learner to control the pace and time it takes to learn the material [49, 50].

### 4.4 Virtual Community of Practice

The findings of this study reported that almost half of the participating students and teachers used SM to network with other dental professionals. SM was found to provide their users with a virtual community of practice (CoP) [51]. A virtual CoP enables learners to interact primarily within a virtual environment, using online communication technologies [52]. As groups interact, they develop a social identity where common ideas, knowledge, concepts, and other social tools become communal properties [51, 53].

### 4.5 Sense of Community

This study demonstrated that SM facilitated the coordination of student and teacher groups, leading to a strong sense of community and, consequently, increased student interactivity [54, 55, 56]. One example of this is students reporting in this study their concerns and doubts expressed on SM about the impact of COVID‐19 on their studies. Similar expressions of student emotions on SM were reported by Hennessy et al. [55].

### 4.6 Elraggal Model for Using SM in Dental Education

The results of this study enabled the development of the Elraggal model for the use of SM in dental education (Figure 2). SM can promote student‐centred learning by developing the learning environment using social, collaborative, problem‐based, active, blended and flipped classroom approaches. Learning activities can also be enhanced through student collaboration, using SM as an information resource, and interaction with dental‐related posts. SM also allowed both dental students and their teachers to feel a sense of community and learn in a virtual CoP.

**FIGURE 2:**
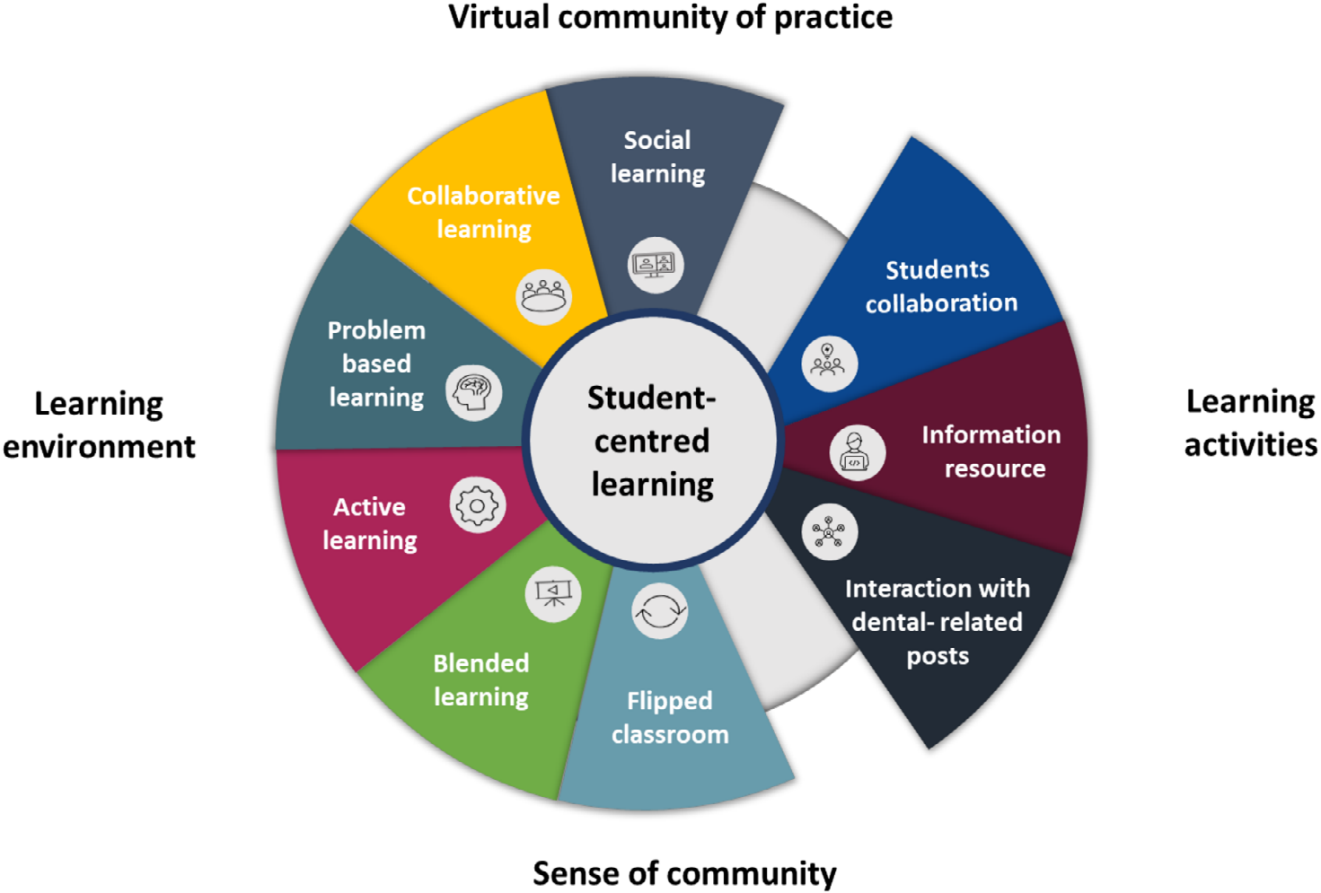
Elraggal model for using social media in dental education.

### 4.7 Barriers to Using SM in Dental Education

Although the study participants recognised the value of SM as a source of information, most expressed concerns about the accuracy of published material. These results were supported by a systematic review of the role of SM in medical education [56] highlighted some concerns from medical students and teachers about the accuracy of the information published on SM, because this information was not peer reviewed as in traditional medical journals.

Most of the participating dental teachers and a smaller number of the dental students expressed concerns regarding their privacy when they used SM for academic purposes. Dental teachers did not want their private SM accounts to be exposed to their students. Also, some of the dental students wanted to keep their private SM accounts separate from their academic life. That is why some dental students and their teachers preferred to maintain communication via email, as a more formal and professional channel. Privacy concerns have been reported in other studies by dental teachers [29], undergraduate dental students [22, 29, 57] and undergraduate medical students [58].

Several undergraduate dental students expressed their concerns regarding the unprofessional behaviour of other students on SM, and the resulting adverse impact on student learning. Sharka et al. [29] found that 38% of undergraduate dental students and teachers were concerned about unprofessional attitudes from fellow students on SM, such as posting patients' pictures online without obtaining their consent, despite General Dental Council (GDC) guidance on the unprofessional use of SM in the UK [59].

Almost half of the interviewed students and their teachers in this study described SM as a distractive tool. Off‐topic activities, such as advertisements, games, and online shopping, were the most distracting events reported by participating dental students outside the classroom. It has been reported that dental students routinely use SM whilst attending lectures, despite believing that this distraction was detrimental to their learning [60]. In a study conducted by Rayan et al. [29], half of the dental students and their teachers were concerned about spending too much time, approximately 2 h daily, on SM.

## 5 Conclusion

The use of SM by dental students for learning purposes has shifted the educational paradigm to a more student‐centred approach where knowledge is socially constructed. Students are given the freedom, space and opportunity to be more proactive and to construct their knowledge through social interactions with their peers within a virtual CoP. SM is potentially helpful and effective educational tools that can supplement classroom teaching and encourage problem‐based, flipped classroom and blended learning approaches.

However, there are some barriers regarding the quality of information published on SM, and some supervision and guidance from their teachers are required to ensure students receive accurate and evidence‐based information. Additional barriers relate to privacy and professionalism, which have resulted in guidelines from the regulators.

## Funding

The authors have nothing to report.

## Conflicts of Interest

The authors declare no conflicts of interest.

## Data Availability

The data that support the findings of this study are available on request from the corresponding author. The data are not publicly available due to privacy or ethical restrictions.
